# Precision approach for transradial access during PCI: a state-of-the-art review

**DOI:** 10.3389/fcvm.2026.1755958

**Published:** 2026-07-21

**Authors:** Tanawat Attachaipanich, Muzamil Khawaja, Hafeez Ul Hassan Virk, Dmitriy N. Feldman, Elmir Omerovic, Ian C. Gilchrist, Arnold H. Seto, Chayakrit Krittanawong

**Affiliations:** 1 Department of Internal Medicine, University of Missouri-Kansas City School of Medicine, Kansas city, MO, United States; 2 Department of Cardiology, Emory University, Atlanta, GA, United States; 3 Division of Cardiovascular Disease, Department of Medicine, Albert Einstein Healthcare Network, Philadelphia, PA, United States; 4 Division of Cardiology, Interventional Cardiology and Endovascular Laboratory, Weill Cornell Medical College, New York-Presbyterian Hospital, New York, NY, United States; 5 Department of Cardiology, Sahlgrenska University Hospital, Gothenburg, Sweden; 6 Geisel School of Medicine at Dartmouth, Section of Cardiovascular Medicine, Heart and Vascular Center, Dartmouth Hitchcock Medical Center, Hanover, NH, United States; 7 Department of Medicine, VA Long Beach Healthcare System, Long Beach, CA, United States; 8 Department of Medicine, Charles R. Drew University of Medicine and Science, Willowbrook, CA, United States; 9 Department of Medicine, University of California, Irvine, CA, United States; 10 HumanX, Delaware, DE, United States

**Keywords:** acute coronary syndromes, percutaneous coronary intervention, radial access, transradial approach, radial artery spasm, radial artery occlusion

## Abstract

The transradial approach (TRA) has become the preferred vascular access for coronary angiography and percutaneous coronary intervention (PCI), offering reductions in bleeding and vascular complications compared with the transfemoral approach, and lower mortality in patients with acute coronary syndromes (ACS). Technical refinements have further enhanced procedural success with TRA. Variations in puncture technique (ultrasound-guidance, through-and-through vs. single-wall technique) and the use of vasodilator “radial cocktails” can improve access success and reduce the risk of radial artery spasm (RAS), while adequate anticoagulation, appropriate sheath sizing, and optimized radial band removal techniques help to reduce radial artery occlusion (RAO). Alternative access strategies such as distal radial (dTRA) and ulnar approaches offer advantages including lower RAO rates and preservation of the proximal radial artery for future conduit use, though these techniques are limited by higher crossover rates, longer cannulation times, and the need for additional operator experience. Optimal intraprocedural anticoagulation in the TRA era remains uncertain, as the bleeding advantage of alternatives to unfractionated heparin may be attenuated given the inherently lower access-site bleeding risk. Procedural complications, including hematoma, RAS, RAO warrant prevention and prompt recognition. Future research should refine anticoagulation strategies, personalize access-site selection, and further evaluate dTRA and ulnar approaches as operator experience matures.

## 1 Introduction

The transradial approach (TRA) has become the preferred vascular access for both coronary angiography (CAG) and percutaneous coronary intervention (PCI) (1). Compared with transfemoral access (TFA), TRA consistently reduces bleeding and vascular complications, lowers mortality among patients with acute coronary syndromes (ACS), and enhances patient comfort through early ambulation and improved overall satisfaction (1–3). Since its first application for CAG in 1989 and subsequent expansion to PCI in 1993, TRA has evolved from a technically challenging procedure with a steep learning curve into a routinely performed and widely adopted access strategy (4, 5).

**Figure 1:**
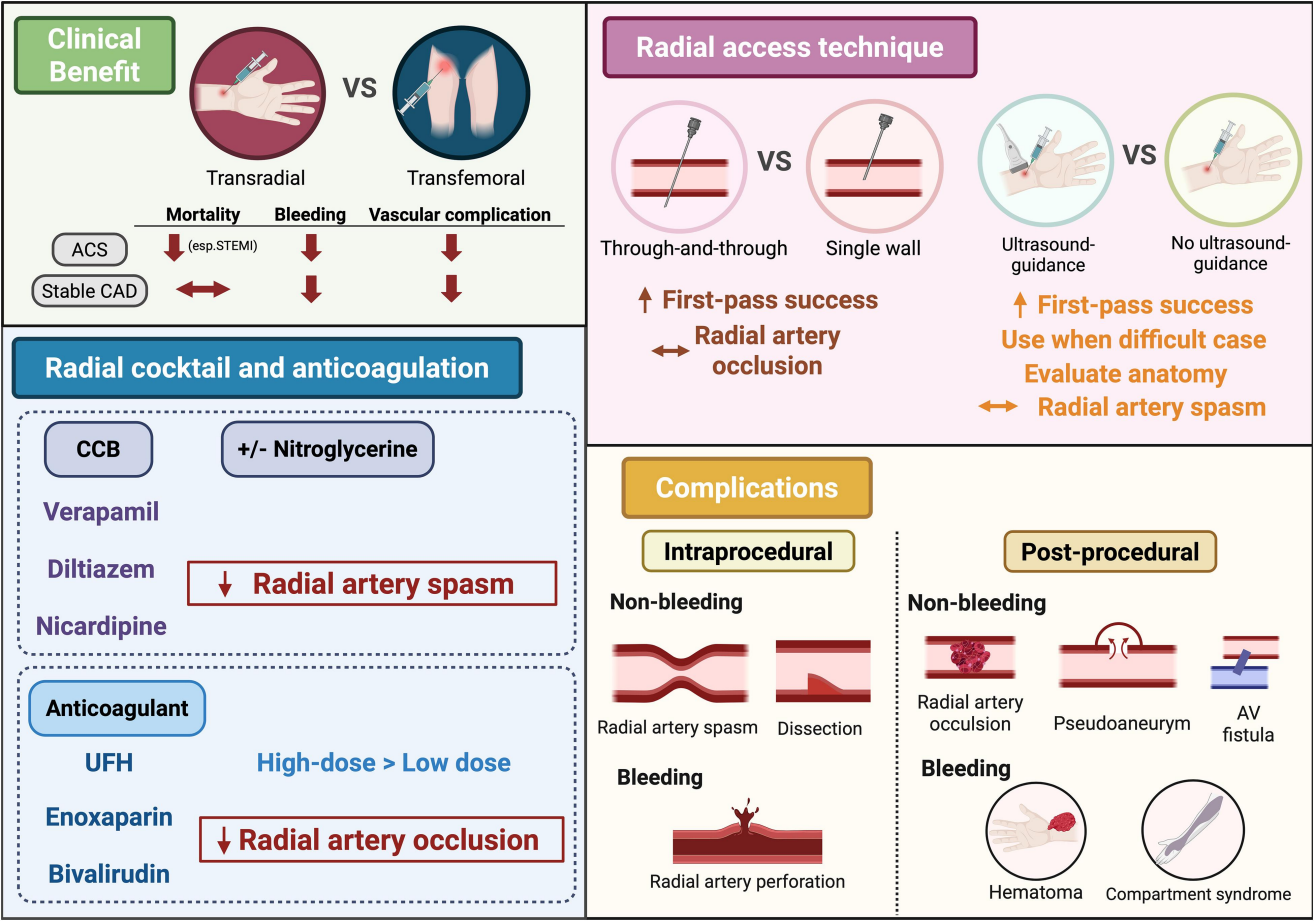
Algorithmic approach to managing transradial access complications. The flowchart outlines the classification and recommended management strategies for these complications. Created in BioRender. Attachaipanich, T. (2026) https://BioRender.com/vp0or3b, licensed under CC BY.

**Central illustration:**
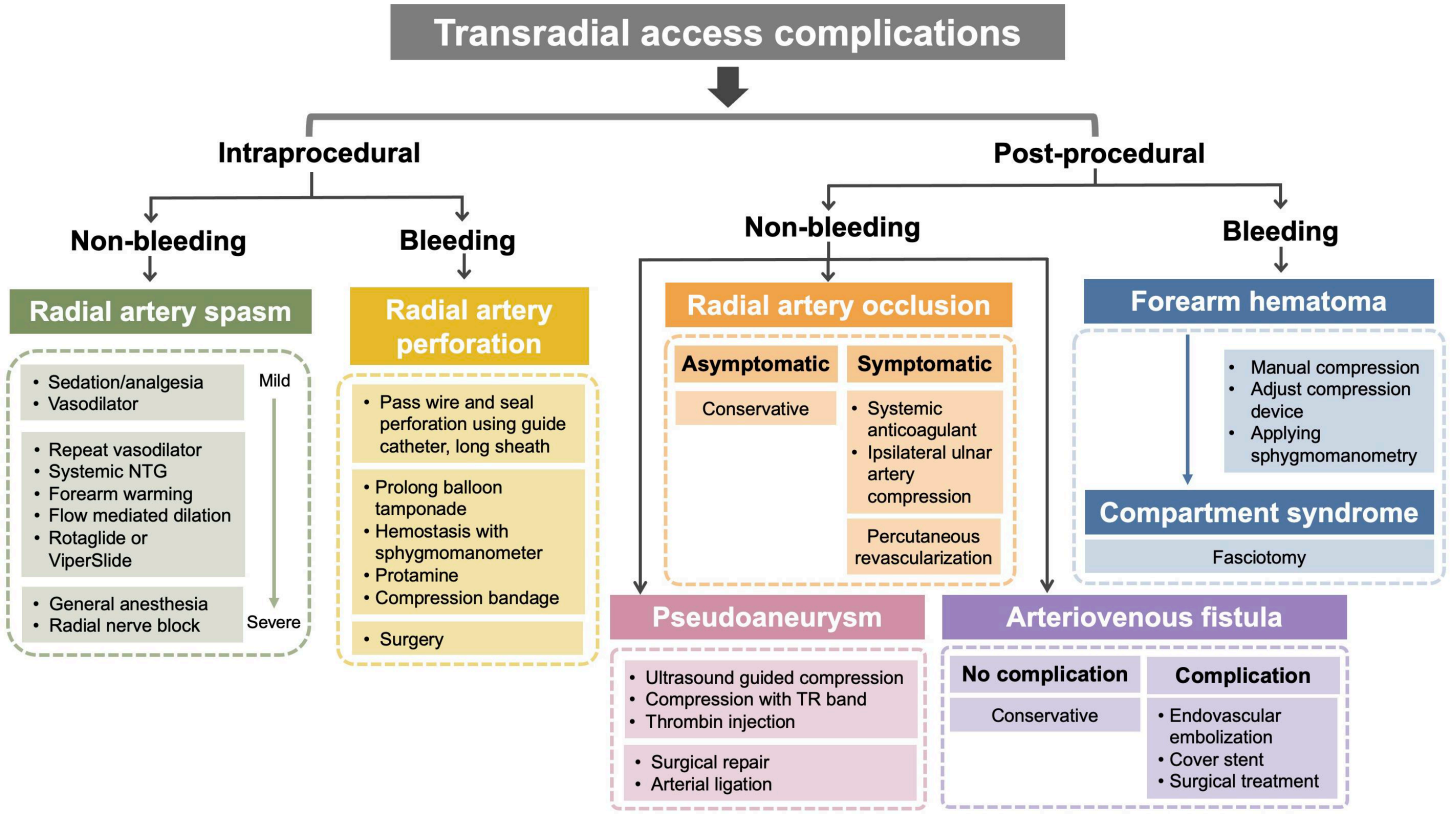
Transradial access: benefits, technique, pharmacology, and complications. Created in BioRender. Attachaipanich, T. (2026) https://BioRender.com/vp0or3b, licensed under CC BY.

In the contemporary PCI era, as ischemic outcomes and survival of ACS patients have improved, attention has increasingly shifted towards minimizing periprocedural complications, particularly bleeding, which is an independent predictor of adverse short- and long-term outcomes (6). Vascular access strategy plays a pivotal role in this regard. The superficial location of the radial artery facilitates direct compression and predictable hemostasis, but its smaller caliber and the presence of anatomical variations can make it more technically challenging.

Robust evidence from multiple large-scale randomized trials and meta-analyses has consistently demonstrated the safety and efficacy of TRA, leading current guidelines to recommend a “radial-first” strategy for cardiac catheterization whenever feasible (1–3). Contemporary registry data further support this paradigm shift, demonstrating earlier and greater adoption of TRA in Europe, with rates exceeding 80% by 2015 in the Swedish SCAAR registry and similarly high utilization reported across Europe, while use in the United States has increased substantially over the past decade, reflecting global convergence toward a radial-first strategy (7, 8). This review summarizes the contemporary evidence on TRA, highlighting technical considerations, adjunctive pharmacology, access-site complications, and future perspectives.

## 2 Radial vs. femoral access

### 2.1 Acute coronary syndromes

Historically, the TFA was the default vascular access for CAG and PCI. Over the past two decades, accumulating evidence from randomized trials and meta-analyses has shifted practice toward TRA. Compared with TFA, TRA consistently reduces bleeding and vascular complications and, in higher-risk ACS populations, has been associated with lower mortality (9–12). Based on large body of evidence, contemporary guidelines have upgraded radial access recommendation for patients with ACS (Class I, Level A) (1).

Early observational data from a large registry of more than 39,000 PCI patients demonstrated that TRA was associated with lower transfusion requirements and reduced 1-year mortality compared with TFA (13). These findings prompted large-scale randomized trials. The RIVAL trial (*n* = 7,021 ACS) demonstrated no significant difference in the primary outcome or major bleeding, but TRA significantly reduced major vascular complications (14). Subgroup analyses of RIVAL trial demonstrated lower mortality and improved primary outcomes among ST-elevation MI (STEMI) patients and in high-volume radial centers, emphasizing the influence of patient risk and operator expertise (9). Although limited by low bleeding event rates, this study highlighted the potential safety advantages of TRA. Subsequent large randomized trials conducted in radial-experienced centers further supported the benefits of TRA. The MATRIX Access trial (*n* = 8,404 ACS) showed that TRA significantly reduced primary composite outcome which was primarily driven by reductions in major bleeding and all-cause mortality (15). Notably, the reduction in bleeding of TRA was confined to access-site events, whereas non-access-site major bleeding did not differ (15).

In STEMI, multiple randomized studies consistently demonstrated reductions in bleeding, vascular complications, and in some trials mortality, establishing TRA as superior to TFA (9–11). A prespecified analysis of MATRIX Access (*n* = 4,010 STEMI), confirmed consistent reductions in ischemic and bleeding outcomes, including mortality, irrespective of ACS presentation (15). In contrast, the SAFARI-STEMI randomized trial (*n* = 2,292 STEMI) reported no significant differences in 30-day mortality or major bleeding between TRA and TFA. However, the trial was stopped early, enrolled lower-risk patients, and used bleeding-avoidance strategies, which likely attenuated differences between groups (16).

For non-ST-segment elevation ACS (NSTE-ACS), there have been no randomized trials exclusively focusing on this population, and most studies did not report outcomes separately for non-ST-segment elevation MI (NSTEMI) and unstable angina (UA). Nevertheless, evidence from large ACS trials with predominance of NSTE-ACS patients support the safety and efficacy of TRA (9, 15). A meta-analysis of 24 studies including 22,843 participants demonstrated that TRA significantly reduced mortality, ischemic events, major bleeding, and vascular complications across all ACS presentations (2). More recent meta-analyses have been consistent with these findings, further supporting the benefit of TRA in ACS (17, 18).

Overall, current evidence strongly supports a radial-first approach across the spectrum of ACS. TRA consistently reduces access-site bleeding and vascular complications, and in high-risk groups such as STEMI, provides a survival benefit, establishing TRA as the default access strategy in contemporary clinical practice. A summary of the major randomized studies comparing TRA and TFA in STEMI and NSTE-ACS populations is presented in Tables 1, 2, respectively.

**Table 1:** Summary of Major randomized clinical trials comparing transradial and transfemoral access in STEMI patients.

Study/RCT name	TRA (n)	TFA (n)	Age	Follow up	Primary endpoint	Mortality	Bleeding	Vascular complication	Ref
RIVAL (STEMI subgroup, 2012)	955	1,003	Radial 60 ± 12.0; Femoral 59 ± 11.7	30 days	Composite of Death, MI, stroke or non-CABG major bleeding at 30 days: 3.1% radial vs 5.2% femoral; HR 0.60 (95%CI 0.38–0.94); *p* = 0.026	1.3% radial vs 3.2% femoral; HR 0.39 (95%CI 0.20–0.76); *p* = 0.006	non-CABG major bleeding 0.8% radial vs 0.9% femoral; HR 0.92 (95%CI 0.36–2.39), *p* = 0.87	1.3% radial vs 3.5% femoral; HR 0.36 (95%CI 0.19–0.70); *p* = 0.002	(9)
RIFLE-STEACS (2012)	500	501	Radial 65 (56–75); Femoral 65 (55–77)	30 days	NACE at 30 days (cardiac death, MI, TLR, stroke, non-CABG bleeding): 13.6% radial vs 21.0% femoral; *p* = 0.003	5.2% radial vs 9.2% femoral; *p* = 0.020	Non-CABG bleeding: 7.8% radial vs 12.2% femoral; *p* = 0.026.	NA	(10)
STEMI-RADIAL (2014)	348	359	Radial 62.7 ± 11.7; Femoral 61.5 ± 11.2	6 months	Major bleeding and access-site complications at 30 days: 1.4% radial vs 7.2% femoral; *p* = 0.0001	30 days: 2.3% radial vs 3.1% femoral, *p* = 0.64; 6 months: 2.3% radial vs 3.6% femoral, *p* = 0.31	Major bleeding: 1.4% radial vs 7.2% femoral; *p* = 0.0001	0.3% radial vs 0.8% femoral; *p* = 0.62	(11)
MATRIX (STEMI subgroup, 2017)	2,001	2,009	Radial 63.7 ± 12.1; Femoral 64.0 ± 12.1	30 days	NACE at 30 days (death, MI, stroke or BARC 3 or 5): 7.2% radial vs 8.3% femoral; RR 0.86 (95%CI 0.69–1.08); *p* = 0.18	2.4% radial vs 2.7% femoral; RR 0.87 (95%CI 0.59–1.29); *p* = 0.49	BARC 3 or 5: 1.9% radial vs 3.0% femoral; RR 0.62 (95%CI 0.41–0.94); *p* = 0.022	Surgical access site repair: 0.2% radial vs 0.6% femoral; RR 0.36 (95%CI 0.12–1.14); *p* = 0.07	(12)
SAFARI-STEMI (2019)	1,136	1,156	Radial 61.6 ± 12.3; Femoral 62.0 ± 12.1	30 days	All-cause mortality at 30 d; 1.5% radial vs 1.3% femoral; HR 1.15 (95% CI 0.58–2.30); *p* = 0.69	1.5% radial vs 1.3% femoral; HR 1.15 (95% CI 0.58–2.30); *p* = 0.69	TIMI non-CABG major or minor: 1.4% radial vs 2.0% femoral; HR 0.71 (95%CI 0.38–1.33), *p* = 0.28	NA	(16)

BARC, Bleeding Academic Research Consortium; CABG, coronary artery bypass grafting; HR, hazard ratio; MI, myocardial infarction; NA, no information; NACE, Net Adverse Clinical Events; RR, risk ratio; STEMI, ST-segment elevation acute coronary syndrome; TFA, transfemoral access; TIMI, Thrombolysis in Myocardial Infarction; TLR, target lesion revascularization; TRA, transradial access.

**Table 2:** Summary of Major randomized clinical trials comparing transradial and transfemoral access in NSTE-ACS patients.

Study/RCT name	TRA (n)	TFA (n)	Age	Follow up	Primary endpoint	Mortality	Bleeding	Vascular complication	Ref
RIVAL (NSTE-ACS subgroup, 2012)	2,552	2,511	Radial 63 ± 11.3; Femoral 63 ± 11.7	30 days	Composite of Death, MI, stroke or non-CABG major bleeding at 30 days: 3.8% vs 3.5%; HR 1.11 (95%CI 0.83–1.48); *p* = 0.491	1.3% vs 0.8%; HR 1.66 (95%CI 0.94–2.92); *p* = 0.082	non-CABG major bleeding 0.6% vs 1.0%; HR 0.66 (95%CI 0.35–1.23), *p* = 0.116	Major vascular access-site complications: 1.5% vs 3.8%; HR 0.38 (95%CI 0.26–0.55); *p* < 0.001	(9)
SAFE-PCI for Women (All randomized, 2014)	893 (52.7% NSTE-ACS)	894 (56.3% NSTE-ACS)	Radial 63.3 (55.1, 72.2); Femoral 63.9 (55.7, 72.0)	30 days	Composite of BARC 2, 3, or 5 bleeding or vascular complications**:** 0.6% radial vs 1.7% femoral; OR 0.32 (95% CI 0.12–0.90), *p* = 0.03	NA	BARC 2, 3, or 5 bleeding: 0.6% radial vs 1.6% femoral	Pseudoaneurysm 0.1% radial vs 0.1% femoral, arterial occlusion 0% all group, arteriovenous fistula 0% all group	(22)
SAFE-PCI for Women (PCI subgroup, 2014)	345 (70.7% NSTE-ACS)	346 (72.5% NSTE-ACS)	Radial 65.1 (56.5, 73.7); Femoral 63.9 (56.5, 72.9)	30 days	Composite of BARC 2, 3, or 5 bleeding or vascular complications**:** 1.2% radial vs 2.9% femoral**;** OR 0.39 (95% CI 0.12–1.27), *p* = 0.12	NA	BARC 2, 3, or 5 bleeding: 1.2% radial vs 2.9% femoral	Pseudoaneurysm 0.3% radial vs 0% femoral, arterial occlusion 0% all group, arteriovenous fistula 0% all group	(22)
MATRIX (NSTE-ACS subgroup, 2017)	2,196	2,198	Radial 67.2 ± 11.3; Femoral 67.5 ± 11.3	30 days	NACE at 30 days (death, MI, stroke or BARC 3 or 5): 12.2% radial vs 14.7% femoral; RR 0.82 (95%CI 0.69–0.97); *p* = 0.023	0.8% radial vs 1.7% femoral; RR 0.50 (95% CI 0.28–0.88); *p* = 0.014	BARC 3 or 5: 1.3% radial vs 1.7% femoral; RR 0.75 (95%CI 0.46–1.23); *p* = 0.26	Surgical access site repair: 0.3% radial vs 0.2% femoral; RR 1.40 (95%CI 0.44–4.40); *p* = 0.57	(12)

BARC, Bleeding Academic Research Consortium; CABG, coronary artery bypass grafting; HR, hazard ratio; MI, myocardial infarction; NA, no information; NACE, Net Adverse Clinical Events; NSTEMI, non-ST-segment elevation acute coronary syndrome; RR, risk ratio; TFA, transfemoral access; TRA, transradial access.

### 2.2 Stable coronary artery disease

In patients with stable coronary artery disease (CAD), randomized trials directly comparing TRA with TFA are limited, with most evidence derived from subgroup analyses of larger studies that included both stable CAD and ACS populations. Current guidelines recommend TRA as the preferred access strategy in stable CAD, primarily due to reductions in bleeding and vascular complications, although no significant differences in mortality or MACE have been seen compared with TFA (19). This position is supported by a subgroup analysis from a meta-analysis of 24 randomized trials. In the stable CAD subgroup, TRA was associated with lower rates of major bleeding and vascular complications, but no significant difference in MACE or mortality (2). Notably, only seven studies contributed stable CAD patients to this analysis, and most included mixed populations of stable CAD and ACS.

Dedicated randomized studies in stable CAD remain limited. In the randomized OCTOPLUS trial (*n* = 377; 47% stable CAD), demonstrated TRA significantly reduced vascular complications compared with TFA (20). A small randomized study similarly showed shorter hospital stays and greater patient preference with TRA (21). The SAFE-PCI for Women randomized study (*n* = 1,787; 45% stable CAD), also demonstrated that TRA reduced bleeding and vascular complications compared with TFA (22). Data from a large retrospective cohort within the National Cardiovascular Data Registry's CathPCI Registry showed a consistent benefit of TRA in reducing mortality, major bleeding, and vascular complications among patients undergoing PCI (23). Although this study included a non-ACS population (33.7%), outcomes were not reported separately for stable CAD (23). More recently, a prospective observational study including only patients with stable CAD found that TRA was associated with significantly lower mortality and major bleeding compared with TFA (24). A summary of the major randomized studies comparing TRA and TFA in stable CAD patients is provided in Table 3.

**Table 3:** Summary of Major randomized clinical trials comparing transradial and transfemoral access in stable CAD patients.

Study/RCT name	TRA (n)	TFA (n)	Age	Follow up	Primary endpoint	Mortality	Bleeding	Vascular complication	Ref
ACCESS (1997)	300 (NA% stable CAD)	300 (NA% stable CAD)	Radial 61 ± 11; Femoral 62 ± 10	1 month	Successful coronary canulation 93% radial vs 99.7% femoral (*p* < 0.001)	0.3% radial vs 0% femoral (*P* = 0.172)	Major entry-site bleeding (Hb loss ≥2 mmol/L, transfusion or vascular repair): 0% radial vs 2.0% femoral	0% radial vs 1.0% femoral	(128)
OCTOPLUS (2004)	192 (NA% stable CAD)	185 (NA% stable CAD)	Radial 82.6 ± 2.7; Femoral 83.0 ± 3.1	In-hospital	Composite vascular endpoint (vascular/bleeding complications): 1.6% radial vs 6.5% femoral (*p* = 0.029)	4.3% radial vs 3.3% femoral	Transfusion: 1% vs 1.6%	Large hematoma 1.6% radial vs 6.5% femoral (*p* = 0.03)	(20)
SAFE-PCI for Women (All randomized, 2014)	893 (46.8% Non-ACS)	894 (43.5% Non-ACS)	Radial 63.3 (55.1, 72.2); Femoral 63.9 (55.7, 72.0)	30 days	Composite of BARC 2, 3, or 5 bleeding or vascular complications**:** 0.6% radial vs 1.7% femoral**;** OR 0.32 (95% CI 0.12–0.90), *p* = 0.03	NA	BARC 2, 3, or 5 bleeding: 0.6% radial vs 1.6% femoral	Pseudoaneurysm 0.1% radial vs 0.1% femoral, arterial occlusion 0% all group, arteriovenous fistula 0% all group	(22)
SAFE-PCI for Women (PCI subgroup, 2014)	345 (28.4% Non-ACS)	346 (27.2% Non-ACS)	Radial 65.1 (56.5, 73.7); Femoral 63.9 (56.5, 72.9)	30 days	Composite of BARC 2, 3, or 5 bleeding or vascular complications**:** 1.2% radial vs 2.9% femoral**;** OR 0.39 (95% CI 0.12–1.27), *p* = 0.12	NA	BARC 2, 3, or 5 bleeding: 1.2% radial vs 2.9% femoral	Pseudoaneurysm 0.3% radial vs 0% femoral, arterial occlusion 0% all group, arteriovenous fistula 0% all group	(22)
RADIAL-CABG (2013)	64 (stable angina 35.9%)	64 (stable angina 46.8%)	Radial: 64.7 ± 6.3 Femoral: 66.7 ± 6.3	In-hospital	Contrast volume used in angiography: 171 ± 72 ml (radial) vs 142 ± 39 mL (femoral), *p* < 0.01)	0% in all group	NA	0% in all group	(129)
Hamilton (2025)	3,784 (100% stable CAD)	2,374 (100% stable CAD)	Radial: 65.4 ± 10.9 Femoral: 65.9 ± 11.8	In-hospital and 5 years	In-hospital major bleeding 0.4% radial vs 0.8% femoral *p* = 0.039	30-day mortality 0.8% radial vs 1.5% femoral, *P* = 0.021, 1-year mortality 3.0% radial vs 4.0% femoral *P* = 0.045, 5-year mortality 20.3% radial vs 21% femoral *p* = 0.65	In-hospital major bleeding 0.4% radial vs 0.8% femoral *p* = 0.039	Vessel dissection 0.2% radial vs 0.2% femoral *p* = 0.64 Pseudoaneurysm 0.1% radial vs 0.7% femoral *p* < 0.001	(130)

ACS, acute coronary syndrome; BARC, Bleeding Academic Research Consortium; CAD, coronary artery disease; Hb, hemoglobin; MI, myocardial infarction; NA, no information; OR, odds ratio; PCI, percutaneous coronary intervention; TFA, transfemoral access; TRA, transradial access.

Overall, current evidence suggests that TRA provides advantages over TFA in patients with stable CAD, primarily through reductions in bleeding and vascular complications and improved patient satisfaction, while mortality and ischemic outcomes remain comparable. However, high-quality large, randomized data specifically dedicated to this population remain limited.

## 3 Radial artery access techniques

### 3.1 Through-and-through approach vs. single-wall approach

Compared with the femoral artery, the radial artery is smaller in caliber, has a thicker muscular wall, and exhibits a narrow lumen-to-wall ratio. These anatomical features make needle stabilization more challenging and predispose to arterial rolling, providing the rationale for distinct puncture techniques (25). Two puncture strategies are commonly used, the through-and-through (double-wall) approach and the single-wall approach, which are often performed using different needle designs and puncture systems, with the double-wall technique typically using a two-piece needle (inner stylet and outer cannula) and the single-wall technique using an open needle without a stylet (3, 25).

In the through-and-through approach, the needle is advanced through both the anterior and posterior walls and then slowly withdrawn until pulsatile blood flow is obtained, thereby facilitating coaxial guidewire passage (25, 26). Unlike femoral artery, where posterior wall puncture carries a significant bleeding risk, bleeding in the radial artery is generally well controlled by vasomotor tone and sheath tamponade. In contrast, the single-wall technique punctures only the anterior wall and has been proposed to reduce intimal trauma. However, in practice most vascular injury results from sheath insertion rather than the puncture itself (25, 26).

A randomized trial of 412 patients directly comparing these two strategies demonstrated that the double-wall approach was faster, more predictable, and associated with higher first-pass success, without an increase in complications including radial artery occlusion (RAO) (25). A subsequent randomized study of 500 patients, however, found no significant differences between the double-wall and single-wall approaches (27). The discrepancies between these trials may reflect differences in operator variability, with a larger number of operators involved in the latter study, as well as procedural complexity, since the first trial included only diagnostic angiography whereas the latter also involved PCI cases. Additionally, the learning curve and cumulative technical experience with TRA may have influenced the outcomes. In practice, both approaches are considered safe and effective, with low rates of RAO and vascular complications. Therefore, the choice of puncture strategy is largely determined by operator preference and experience, although the double-wall approach is often favored for its efficiency (3, 25).

### 3.2 Ultrasound guidance vs. no ultrasound guidance

Obtaining arterial access can be technically challenging, particularly in patients with weak pulses, hypotension, cardiogenic shock, or when ulnar artery access is required (3). The radial artery is small in caliber and may be diminutive, collapsible, calcified, or highly mobile. In addition, anatomical variations such as high bifurcations, loops, or tortuosity are present in up to 10% of patients, further contributing to puncture failure (28, 29).

Ultrasound guidance has been shown to improve the success of radial artery cannulation compared with palpation alone. In the randomized RAUST trial (*n* = 698), real-time ultrasound guidance significantly reduced time to access, increased first-pass success, and decreased the number of difficult punctures compared to palpation. However, no significant differences were observed in the rates of radial artery spasm (RAS), patient-reported pain, or bleeding (29).

Beyond facilitating puncture, preprocedural ultrasound provides valuable information regarding vessel size, calcification, and occlusion, and can identify anatomical variants that predispose to procedural failure (28). Current expert consensus recommends that operators be proficient in ultrasound guidance to improve successful vascular access, and that real-time ultrasound should be available and used when difficult access is encountered (30). Ultrasound can also guide sheath selection, helping to avoid an unfavorable sheath-to-artery diameter ratio, which increases the risk of both RAS and RAO. A previous prospective study demonstrated that a lower ratio between the radial artery inner diameter and sheath outer diameter (<1) was associated with a higher incidence of severe radial flow reduction after transradial access (31).

### 3.3 Routine wrist/forearm angiography

Routine wrist/forearm angiography after sheath insertion has been proposed as an adjunctive strategy to define forearm arterial anatomy before wire or catheter advancement (32). This approach may allow early recognition of radial loops, severe tortuosity, high radial artery origin, stenosis, severe kinking, or spasm, which can contribute to procedural difficulty, access failure, and vascular complications. In a single-center observational study of STEMI patients, 762 patients treated before implementation of routine preprocedural radial angiography were compared with 4,306 patients treated after its adoption (33). Routine radial angiography identified radial artery anomalies in 7.6% of patients and was associated with significantly reduced crossover from primary TRA (4.6% to 3.1%), lower access-site bleeding complications (6.8% to 4.3%), and shorter procedural duration, without increasing contrast volume or fluoroscopy time (33). However, because available data are largely observational and derived from experienced radial centers, its generalizability to less experienced radial centers remains uncertain. Randomized evidence demonstrating the clinical benefit of routine wrist or forearm angiography remains limited. Therefore, routine or selective forearm angiography, particularly in high-risk patients or when difficult anatomy is suspected or resistance is encountered, may be considered as part of a precision transradial strategy, while future prospective studies are needed to define its optimal use.

## 4 Radial cocktails

Because of its small caliber, the radial artery is particularly prone to RAS, one of the most common complications of TRA. RAS is characterized by sudden arterial constriction that limits catheter manipulation and causes patient discomfort (34). To mitigate this risk, a “radial cocktail” consisting of vasodilators and anticoagulants is typically administered after sheath insertion and, when necessary, during catheter exchanges or prior to sheath removal (3, 35).

Intra-arterial vasodilators have been shown to increase radial artery diameter and reduce the incidence of RAS (36). Commonly used agents include calcium channel blockers such as verapamil (2.5–5 mg), diltiazem (2.5–5 mg), or nicardipine (250–500 μg), as well as nitroglycerin (100–200 μg) (3). Both drug classes significantly reduce the risk of RAS, and additive benefits have been observed when used in combination. A meta-analysis of 22 studies reported that verapamil 5 mg, either alone or combined with nitroglycerin, provides the most effective protection against RAS (37). Although generally safe, vasodilators should be used cautiously in patients who are hemodynamically unstable, have right ventricular infarction, or severe left ventricular dysfunction (3). Alternative routes of nitroglycerin, such as topical or subcutaneous, have also been shown to increase radial artery diameter and reduce RAS with fewer systemic side effects (38).

Verapamil is the most extensively studied intra-arterial spasmolytic agent for TRA and may also be administered immediately before sheath removal to prevent RAS, facilitate catheter or sheath extraction, and reduce patient discomfort (3). The SPASM randomized trial (*n* = 1,219) demonstrated that intra-arterial verapamil significantly lowered the incidence of RAS compared with placebo (35). Several randomized studies have further shown that verapamil increases radial artery diameter throughout the vessel course while reducing the frequency of RAS (36, 39). In addition, a prospective study found that a combination of verapamil and nitroglycerin reduced the maximum pullback force required during catheter withdrawal, consistent with easier and less traumatic sheath removal, thereby supporting its prophylactic use when RAS is anticipated (40). More recently, a randomized trial comparing intra-arterial nicardipine with verapamil during TRA showed that nicardipine caused significantly less patient discomfort while maintaining similarly low rates of RAS, suggesting it may be a well-tolerated alternative spasmolytic agent (41).

Taken together, current evidence supports the routine use of intra-arterial vasodilators, most commonly verapamil with or without nitroglycerin, in combination with unfractionated heparin, regardless of baseline oral anticoagulant therapy, as part of a standard “radial cocktail” (3). This strategy reduces RAS, improves procedural success, and prevents RAO without significantly increasing bleeding risk.

## 5 Proximal radial vs. distal radial vs. ulnar access

Current guidelines recommend the TRA as the standard access for coronary procedures, with distal radial access (dTRA, or “snuffbox”) and ulnar approach as acceptable alternatives (3, 19). Among these options, conventional proximal radial access (pTRA) is the most established, while dTRA and transulnar access have been increasingly studied in recent years (3, 30, 42).

The dTRA offers an anatomical advantage, as the puncture site lies distal to several anastomotic branches. In the event of an occlusion at the distal site, antegrade radial flow in the forearm can be preserved, minimizing the risk of hand ischemia (42). Randomized studies and meta-analyses have shown that dTRA reduces forearm RAO and large hematomas compared with pTRA. These benefits, however, come at the cost of longer cannulation time, more puncture attempts, and higher crossover rates. Most of these trials were conducted in patients with stable CAD, where additional access time is less critical than in ACS (43, 44). Notably, the prolonged puncture and sheath insertion times can be largely mitigated by ultrasound guidance and increasing operator experience. Beyond RAO prevention, dTRA offers practical advantages, including improved patient comfort, easier hemostasis, and preservation of the proximal radial segment for potential future conduit use (30). A recent network meta-analysis of 47 randomized trials including nearly 39,000 patients confirmed that both dTRA and ulnar access significantly reduced bleeding and hematoma compared with TFA. Moreover, dTRA was associated with a lower risk of RAO compared with pTRA, albeit with a higher rate of access-site crossover (45).

The ulnar artery is another potential alternative when TRA is not feasible. However, its deeper anatomical location, less palpable pulse, and proximity to the ulnar nerve make puncture and hemostasis more challenging. In a randomized trial (*n* = 902), enrollment was terminated early due to high crossover rates in the ulnar group (46). Among patients with successful access, the ulnar approach was non-inferior to TRA for MACE and vascular complications at 60 days, although procedures were performed by operators with limited ulnar experience and without ultrasound guidance (46). Similarly, a meta-analysis of five randomized trials further confirmed that transulnar access has comparable efficacy and safety to TRA but requires more puncture attempts and is associated with higher crossover rates (47). Current guidelines support the ulnar approach only as an alternative when TRA is unavailable or occluded, emphasizing the role of ultrasound to improve procedural success (3, 30).

In practice, pTRA remains the default strategy for most cases; dTRA is preferred when radial artery preservation or RAO prevention is a priority, while ulnar access should be reserved for cases where TRA is not feasible, ideally performed by experienced operators with ultrasound guidance.

## 6 Right radial vs. left radial

The majority of TRA procedures are performed via right radial access (RRA), largely due to the standard configuration of catheterization laboratories and operator position preference. Left radial access (LRA) is less frequently used, but randomized trials and meta-analyses consistently demonstrate that both approaches achieve high procedural success with similarly low rates of complications, including access-site complications and stroke (48, 49). A meta-analysis of randomized studies showed that LRA was associated with modest reductions in fluoroscopy time and contrast use compared with the RRA, likely reflecting the more direct anatomical alignment of the left radial and subclavian arteries with the ascending aorta (48). These benefits are particularly relevant in patients prone to right-sided tortuosity, including elderly individuals, women, shorter stature, or longstanding hypertension, with LRA often providing a more direct path to the aorta (50).

LRA is also advantageous for selective engagement of the left internal mammary artery (LIMA) graft after bypass surgery (3). Evidence regarding radiation exposure has been mixed (51–53). Some studies report no difference in fluoroscopy time or dose between LRA and RRA, while others suggest inconsistent differences in operator exposure depending on laboratory configuration and the use of adjunctive shielding (51–53). Importantly, crossover rates, RAO, and overall procedural complications are comparable between the two approaches (51–53).

Current guidelines recommend a radial-first strategy but do not favor one side over the other (3, 19). Instead, access site selection should be individualized based on patient anatomy, comorbidities, and procedural goals. Preprocedural assessment should consider factors such as subclavian or aortic tortuosity, and graft targets, while ultrasound guidance can further improve success and safety with either approach (3, 19).

## 7 Intraprocedural anticoagulation

### 7.1 Acute coronary syndromes

During PCI, the prothrombotic state driven by thrombin generation and platelet activation makes anticoagulation essential to prevent acute thrombus formation. Unfractionated heparin (UFH) remains the standard anticoagulant during PCI for ACS (1). Its advantages include rapid onset, short half-life, and reversibility with protamine, although limitations include variable pharmacokinetics and the risk of heparin-induced thrombocytopenia (HIT). While no randomized trial has directly compared heparin with placebo in ACS, one randomized study in stable CAD (the CIAO trial) has been conducted (54). Its efficacy in preventing ischemic complications has been established through extensive clinical use and consistently reinforced by its role as the comparator in large randomized trials of newer anticoagulants (55, 56).

Bivalirudin has been recommended as an alternative to UFH in ACS, particularly for patients at high bleeding risk or with HIT (1). Earlier randomized trials in ACS patients comparing bivalirudin with UFH plus routine glycoprotein IIb/IIIa inhibitor (GPI) demonstrated reduced bleeding with bivalirudin, although ischemic outcomes were similar (57–59). However, in the TRA, where baseline bleeding risk is lower, the relative bleeding advantage of bivalirudin appears less pronounced. The single-center randomized HEAT-PPCI trial (*n* = 1,829 STEMI; 80% TRA) found no bleeding benefit of bivalirudin compared with UFH, but reported higher rates of stent thrombosis with bivalirudin (60). Other large randomized trials, including MATRIX (*n* = 7,213 ACS; 50% TRA) and VALIDATE-SWEDEHEART (*n* = 6,006 ACS; 90% TRA) showed no differences in ischemic or bleeding outcomes between UFH and bivalirudin (61, 62). A meta-analysis of 8 randomized studies including 27,491 ACS patients demonstrated that bivalirudin reduced major bleeding in patients undergoing TFA, but conferred no benefit in TRA, where bleeding complications are inherently lower (63). Together, these findings suggest that the advantage of bivalirudin is attenuated in the context of routine TRA.

Low-molecular-weight heparin (LMWH), particularly enoxaparin, has also been shown as an effective alternative to UFH in ACS. In the randomized ATOLL trial (*N* = 910 STEMI; 65% TRA), enoxaparin was not superior to UFH for the primary composite endpoint of death, MI, procedural failure, or major bleeding (64). Randomized trials, including OASIS-5 and OASIS-6, demonstrated that fondaparinux provided ischemic protection comparable to enoxaparin with significantly less bleeding but higher rates of catheter-related thrombosis (65, 66).

In summary, UFH remains the standard anticoagulant of choice during PCI for ACS, with bivalirudin and enoxaparin serving as alternatives in selected patients, though their advantages are diminished in the TRA where baseline bleeding risk is relatively low.

### 7.2 Stable coronary artery disease

Evidence supporting the use of parenteral anticoagulation during elective PCI for stable CAD is limited, particularly in the setting of TRA. The CIAO randomized trial (*n* = 700 stable angina; 100% TFA) found no significant difference in ischemic outcomes between UFH with placebo during elective PCI, while placebo was associated with fewer bleeding events (54). However, this was a small, single-center trial conducted in very low-risk patients, limiting its generalizability. Despite these findings, intraprocedural anticoagulation remains standard best practice. LMWH has been evaluated as an alternative to UFH. In the randomized STEEPLE trial, intravenous enoxaparin significantly reduced major bleeding compared with UFH, with similar efficacy in elective PCI performed via TFA (67). These findings suggest that enoxaparin may offer a superior safety profile; however, evidence in the TRA setting remains limited. Bivalirudin has also been studied in elective PCI. Large randomized studies which included patients with elective PCI showed no difference in ischemic outcomes between bivalirudin and UFH, but major bleeding was significantly lower with bivalirudin (68, 69). These trials suggest that bivalirudin is a reasonable alternative to UFH +/- GP IIb/IIIa inhibitors in elective PCI, particularly in patients at high bleeding risk, although it does not result in superior ischemic outcomes. Importantly, both trials were conducted predominantly via TFA (with high use of GP IIb/IIIa agents), and the bleeding advantage of bivalirudin is likely attenuated in the TRA setting.

Overall, for elective PCI in stable CAD, particularly via TRA, weight-based UFH with activated clotting times (ACT)-guided dosing remains the standard of care. Enoxaparin and bivalirudin are acceptable alternatives.

### 7.3 STEMI after fibrinolysis

In patients with STEMI treated with fibrinolysis, parenteral anticoagulation is recommended by current guidelines for the duration of hospitalization (up to 8 days) or until revascularization is performed, in order to reduce ischemic complications (1). Early fibrinolytic-era randomized trials, such as GUSTO and ASSENT-3, routinely administered UFH for at least 48 h (70, 71). The randomized ASSENT-3 and ExTRACT-TIMI 25 trials demonstrated that enoxaparin reduced ischemic complications compared with UFH, although this benefit was offset by higher major bleeding rates (71, 72). Importantly, in a subset of patients who subsequently underwent PCI, enoxaparin significantly reduced 30-day death or MI, without excess major bleeding (72). Fondaparinux also reduced death and reinfarction in the randomized OASIS-6 trial, particularly in those treated with streptokinase (66). These studies were largely performed via TFA, and evidence for TRA is limited. Given its lower bleeding risk, TRA may enhance the net benefit of enoxaparin or fondaparinux, but further dedicated studies are needed.

## 8 Ideal ACT target

Because UFH has a relatively narrow therapeutic window, balancing the risks of both ischemic and hemorrhagic complications is essential, and ACT monitoring has become the most widely used method for intraprocedural titration in the catheterization laboratory (3, 73). Current guidelines recommend ACT targets based on the concomitant antithrombotic regimen rather than the access site. When UFH is combined with a GPI, a targeted ACT of 200–250 s is recommended. When UFH is used alone, the target is 250–300 s using HemoTec or 300–350 s using Hemochron (3, 73). Importantly, these recommendations are derived largely from small studies, and no randomized trial has prospectively compared the efficacy and safety of ACT-guided vs. fixed-dose regimens.

The relationship between ACT values and clinical outcomes remains inconsistent. A meta-analysis of four randomized trials including nearly 10,000 patients found that ACT did not correlate with ischemic complications and showed only a modest association with bleeding, primarily minor bleeding (74). Lower ACT values did not appear to compromise ischemic protection, while higher values were associated with increased bleeding (74).

More recent registry data have highlighted the impact of vascular access on the relationship between ACT and outcomes. In a retrospective study of 14,637 PCI patients treated with UFH, higher maximal ACT was strongly associated with major bleeding in the TFA group (particularly ACT >290 s), but not in the TRA group (75). ACT was not associated with in-hospital ischemic complications in either access group (75). These findings suggest that during TRA PCI, more intense anticoagulation can be tolerated (including GP IIb/IIIa agents and cangrelor) because TRA substantially reduces access-site bleeding, whereas in TFA the margin for excess anticoagulation is narrower. Nonetheless, non-access-site bleeding remains clinically relevant, particularly in ACS, and continues to correlate with adverse long-term outcomes irrespective of access strategy (76).

Overall, ACT monitoring remains essential for UFH titration. Numeric ACT targets do not differ by access site, but the widespread adoption of TRA may reduce bleeding risk at any ACT level. This access-related protection likely explains why higher ACT levels are less strongly linked to bleeding in TRA compared with TFA, supporting the concept that TRA improves the safety margin of anticoagulation without changing the therapeutic ACT range itself.

## 9 Radial band management

### 9.1 Compression time

Compression devices for achieving hemostasis after sheath removal in TRA are being adopted more widely across the world and remain the predominant method in the US (77). While premature removal and inexperienced staff increase the risk of hematoma, prolonged compression raises the risk of RAO. There is no universal consensus on the optimal duration of compression, though contemporary practice typically involves approximately 60 min of compression after diagnostic angiography and 120–180 min following PCI (3).

Early studies suggested that shorter compression times reduce RAO. In a retrospective cohort of 400 PCI patients managed with a TR Band, a 2-hour protocol significantly lowered RAO compared with conventional 6-hour protocols (5.5% vs. 12% at 24 h; 3.5% vs. 8.5% at 30 days), without increasing bleeding (78). Similarly, the CRASOC series demonstrated that shorter, mild compression (1.5 h) reduced RAO to 2.3% compared with 9.4% using stronger, 4-hour compression. However, shorter duration was associated with higher bleeding and recompression rates (8.4% vs. 1.2%) (79).

A network meta-analysis of 10 randomized trials including over 4,900 patients confirmed that a 2-hour TR Band protocol provides the best balance of safety and efficacy, lowering RAO without increasing hematoma or rebleeding (80). Durations shorter than 90 min consistently increased bleeding, while longer protocols offered no additional benefit (80). In a randomized trial of 129 patients, an accelerated protocol with band deflation initiated 20 min after sheath removal was compared with a regimen adjusted for anticoagulation (30–120 min). Accelerated removal significantly shortened hemostasis time (113 vs. 131 min) without increasing RAO or hematoma, supporting the feasibility of earlier removal under close monitoring (81).

The choice of access site may also influence hemostasis time. The dTRA has been associated with shorter hemostasis duration compared with conventional TRA. In the randomized DISCO RADIAL trial (*n* = 1,307), dTRA achieved a significantly shorter median hemostasis time (153 vs. 180 min), without an increase in bleeding or vascular complications, despite similar rates of RAO between groups (44). These findings suggest that dTRA may facilitate earlier device removal.

Current evidence strongly supports shorter compression duration (≤120 min) as the optimal strategy to reduce RAO without increasing bleeding. Accelerated or expedited weaning protocols may also be appropriate in carefully selected low-risk patients with close monitoring.

### 9.2 Statseal

While shorter compression times reduce the risk of RAO, they must be balanced against the risks of rebleeding, hematoma, and rarely pseudoaneurysm. To address these challenges, adjunctive topical agents have been studied to facilitate earlier and safer deflation. StatSeal, a hemostatic patch composed of a hydrophilic polymer and potassium ferrate, accelerates clot formation by concentrating blood solids. Its mechanism is independent of the coagulation cascade, making it an effective adjunct when applied beneath a radial band (29, 82).

Clinical studies consistently demonstrated that StatSeal shortens compression times without increasing vascular complications. In the pilot randomized STAT study, StatSeal significantly reduced time to complete band removal compared with radial band alone (43 min vs. 160 min) (29). The larger randomized STAT2 trial (*n* = 443) confirmed these findings, showing significantly shorter mean compression times with (66 min vs. 113 min), fewer rebleeding episodes requiring reinflation (0% vs. 67.7%), and lower rates of hematoma, with no difference in RAO (0.4% vs. 0.9%) (82).

The randomized ARCH trial (*n* = 2,114) provided robust evidence supporting StatSeal use (83). Patients were randomized to conventional compression for 120 min, conventional compression for 60 min, or StatSeal-assisted compression for 60 min. Rebleeding occurred in 62% of patients with 60-minute conventional compression and 50% with 120-minute compression, compared with only 5.2% with StatSeal. Median hemostasis time was significantly shorter with StatSeal (72 min vs. 136 and 166 min), without an increase in hematoma or RAO. Delayed discharge due to prolonged radial site care was also significantly reduced in the StatSeal group (83).

These findings confirm that StatSeal facilitates safe and effective early radial band deflation as early as 60 min without an increased risk of hematoma or RAO. In contrast, early deflation without adjunctive support is often associated with rebleeding, reinflation, and prolonged compression times. Adjunctive hemostatic patches therefore represent an effective strategy to accelerate hemostasis after TRA, particularly in patients with high bleeding risk or increased susceptibility to RAO.

### 9.3 Air amount

The optimal volume of air required for radial band inflation remains uncertain and is not specified in published device instructions for use. Device protocols typically recommend an initial inflation of 15–18 mL, followed by staged deflation. However, growing evidence suggests that preservation of antegrade radial flow, rather than the absolute inflation volume, is the key determinant of outcomes.

In retrospective cohort study (*n* = 111), higher inflation volumes (18 mL) were associated with significantly increased rates of RAO compared with lower volumes (12 mL), although RAO remained rare in both groups (84). This suggests that excessive inflation pressure may predispose to RAO. Similarly, a study of 84 patients using an air-titration technique, initial inflation to 2 mL above the bleeding point, followed by gradual down-titration, reported very low RAO rates when inflation was adjusted to the minimal volume required for hemostasis. These findings highlight the importance of individualized inflation strategies to reduce complications (85).

A meta-analysis of 11 randomized studies including nearly 4,700 patients found no significant difference in RAO or bleeding between reduced-air and standard (15 mL) inflation, but higher discomfort with reduced-air strategies (86). The large randomized PROTHECT trial compared different starting volumes (8 mL in the StatSeal group vs. 18 mL in the conventional patent hemostasis group) and found similarly low RAO rates across strategies, reinforcing that maintaining patent hemostasis, rather than using a fixed inflation volume, is the key determinant of vascular outcomes (87).

Overall, an initial inflation of approximately 15 mL remains a reasonable standard. Subsequent titration to the lowest volume that maintains patent hemostasis provides the best balance between effective hemostasis, patient comfort, and long-term vascular safety.

Importantly, hemostasis strategies differ between dTRA and conventional TRA. In technical descriptions of dTRA, lower inflation volumes are required due to the superficial location of the artery overlying bony structures, typically around 3–5 mL, with compression maintained for 2–3 h followed by gradual deflation (88, 89).

### 9.4 Patent hemostasis vs. occlusive techniques

Patent hemostasis is one of most important modifiable postprocedural factors for preventing RAO. It is achieved by applying just enough pressure to prevent bleeding at the puncture site while preserving antegrade radial flow, thereby avoiding complete vessel collapse and distal flow cessation. In contrast, occlusive compression promotes thrombus formation and is a strong predictor of RAO (90).

The PROPHET randomized trial (*n* = 436) demonstrated that patent hemostasis significantly reduced both early (<24 h) RAO (5% vs. 12%) and late (30-day) RAO (1.8% vs. 7%) compared with occlusive compression (90). Similarly, the randomized RACOMAP trial (*n* = 351), which used pneumatic compression guided by mean arterial pressure to ensure ongoing radial flow, reported lower RAO rates with patent hemostasis than with occlusive compression (1.1% vs. 12%) (91).

Adjunctive strategies have been investigated to further improve outcomes. Ipsilateral ulnar artery compression augments radial flow and promotes vasodilator release, facilitating recanalization in patients with acute RAO after TRA (92). In the PROPHET II randomized trial (*n* = 3,000), the addition of prophylactic ulnar artery compression during patent hemostasis further reduced RAO, lowering early RAO from 4.3% to 1% at 24 h and late RAO from 3% to 0.9% at 30 days (90, 93).

Despite these benefits, achieving true patent hemostasis requires significant nursing resources, with repeated plethysmographic or oximetric checks and frequent adjustments of compression to ensure vessel patency. Consequently, inadvertent occlusive hemostasis during patent protocols has been reported in 5%–25% of cases. The use of prophylactic ulnar compression has been shown to increase the success rate of true patent hemostasis to over 95% (94).

The randomized PROTHECT trial (*n* = 1,469) compared three contemporary non-occlusive strategies including traditional patent hemostasis with 2-minute ulnar compression, patent hemostasis with 1-hour ulnar compression, and facilitated hemostasis with a StatSeal disc. At 24 h, RAO rates were low across all groups (3.6%, 5.5%, and 4.7%, respectively), and further declined to 1%–2% by 30 days, with no significant differences between strategies (87).

Overall, patent hemostasis, particularly when combined with adjunctive ulnar compression, is superior to occlusive techniques and remains one of the most effective strategies to minimize RAO after TRA.

## 10. Risk factors, diagnosis, and management of radial access complications

Although TRA significantly reduces bleeding and vascular complications compared with TFA, TRA still has a risk of complications (11, 14, 15). Complications can be broadly categorized by timing, as intraprocedural or postprocedural, and by mechanism, as bleeding-related or non–bleeding-related. Intraprocedural bleeding complications include radial artery perforation, while non-bleeding complications include RAS. Postprocedural bleeding events most often present as hematomas and compartment syndrome, whereas non-bleeding complications include RAO, pseudoaneurysm, arteriovenous fistula (AVF), nerve injury, and infection (34). Although the overall incidence of serious complications is very low, prompt recognition and timely management are essential to prevent progression and avoid long-term morbidity. An algorithmic approach to the recognition and management of TRA complications is summarized in Figure 1.

### 10.1 Hematoma

The reported incidence of forearm hematoma after TRA is 1%–3%, which is substantially lower than with TFA (3, 9, 11, 14). However, systematic postprocedural ultrasonography, such as in the SPIRIT of ARTEMIS study, detected local hematomas in up to 23% of patients undergoing diagnostic angiography, the majority of which were clinically insignificant (95). Owing to the superficial location of the radial artery overlying the radius, hematomas are usually compressible and most hematomas are localized and self-limited. Nonetheless, large forearm hematomas can extend proximally and, if unrecognized, may progress to compartment syndrome and ischemic injury. The key is to educate staff on how to recognize, monitor, and manage hematoma control effectively. If recognized and managed promptly, hematomas rarely progress to compartment syndrome.

Risk factors for hematoma include radial artery perforation, misplacement or inadequate inflation of the compression device, and concomitant treatment with GPI (34, 96, 97).

Diagnosis is primarily clinical, based on forearm swelling, pain, and bruising. Duplex ultrasonography may be used to assess hematoma extent but rarely detects active bleeding, as the source is usually a small branch vessel (34). The EASY classification is frequently used to grade hematomas by size and extent: grade I (≤5 cm, superficial), grade II (≤10 cm with muscular infiltration), grade III (>10 cm below the elbow with muscular infiltration), grade IV (extending above the elbow), and grade V (associated with ischemic threat such as compartment syndrome) (98).

Management is typically conservative. Minor hematomas resolve with continued or repositioned compression. Larger hematomas may be managed by applying a blood pressure cuff inflated to systolic pressure just proximal to the site, with gradual release over 1–2 h, or with repositioned pneumatic compression devices (34). Surgical evacuation is rarely required. Prognosis is generally favorable, provided hematomas are recognized early and treated promptly, thereby preventing progression to compartment syndrome (34). It is crucial to educate nursing staff and catheterization lab technicians to recognize and properly handle compression devices, including repositioning them as needed, to prevent compartment syndrome.

### 10.2 Perforation

Radial artery perforation is a rare complication of TRA, with an incidence of <1% (34, 99). Although uncommon, it can result in serious bleeding and, if unrecognized or inadequately managed, may progress to compartment syndrome requiring surgical intervention. Perforation most often occurs when a guidewire inadvertently enters and ruptures a small side branch, or from the “razor effect” of a catheter tip cutting into the arterial wall during advancement (99). Risk factors include tortuous or anomalous anatomy, small vessel caliber, female sex, short stature, aggressive or repeated wire manipulation, over-anticoagulation, and the use of hydrophilic wires (32, 34, 97).

Diagnosis is usually made intraprocedurally. Patients may experience sudden forearm pain or a vasovagal reaction during wire or catheter advancement (99). Angiography typically confirms the complication by demonstrating contrast extravasation, while Doppler ultrasound may be used to localize the bleeding site and confirm resolution after treatment (32, 34).

Most cases can be managed conservatively, and surgical repair is rarely required (34). Preventive strategies include the use of soft-tipped or polymer-jacketed J-wires, avoidance of forceful wire advancement, early angiography when resistance is encountered, and techniques such as balloon-assisted tracking in patients with challenging anatomy (34).

Management depends on wire position at the time of perforation. If a wire or catheter lies across the perforation, hemostasis can usually be achieved by advancing a long sheath or guide catheter to tamponade the site, allowing completion of the planned intervention (34). If bleeding persists, adjunctive measures such as prolonged balloon tamponade, protamine administration, external compression with a blood pressure cuff inflated to systolic pressure, or compression bandaging may be necessary. If the wire is not positioned across the site, attempts should be made to rewire the vessel proximally to enable sealing strategies (34).

### 10.3 Radial artery spasm

RAS is a relatively frequent complication of TRA, with reported incidence ranging from 4% to 20%. RAS is defined as a temporary, sudden narrowing of the radial artery due to increased vasoreactivity (34, 100). The radial artery is particularly prone to spasm because of its abundance of *α*-adrenergic receptors, which, when stimulated by local trauma, catheter manipulation, or vessel stretching, trigger smooth muscle contraction and vasoconstriction (34).

Diagnosis is usually clinical and may be confirmed angiographically during the procedure (34, 100). Patients typically present with forearm pain, often exacerbated by catheter or sheath movement, accompanied by resistance to wire or device advancement. Damping of arterial pressure tracings and loss of radial pulse may also be observed (34, 100). Severe RAS can necessitate crossover to an alternative access site.

Both patient- and procedure-related factors contribute to RAS risk. Patient-related predictors include female sex, younger age, low body mass index, small radial artery diameter, anatomical variants such as high takeoff, anxiety, smoking, diabetes mellitus, dyslipidemia, and prior coronary artery bypass graft surgery (32, 34, 97, 101). Procedural risk factors include multiple puncture attempts, prolonged or technically difficult catheterization, large sheath-to-artery ratios, frequent catheter exchanges, higher contrast volume, and inadequate analgesia or sedation (32, 34, 97, 101).

Prevention strategies are crucial. Routine use of sedation and intra-arterial vasodilatory cocktails, most commonly verapamil with or without nitroglycerin, is recommended to reduce spasm by attenuating vasoconstriction and improving vessel compliance (34). Hydrophilic-coated sheaths reduce frictional resistance, leading to significantly lower spasm rates compared with uncoated sheaths, and smaller sheath sizes are preferred whenever feasible (102). Additional preventive measures include adequate analgesia to mitigate sympathetic-mediated vasoconstriction driven by patient anxiety and pain, avoidance of diminutive target vessels, and reducing catheter manipulation or exchanges to reduce mechanical stimulation (34). In a randomized study of over 2,000 patients, procedural sedation with analgesia reduced the incidence of RAS from 8.3% to 2.6% and significantly decreased access-site crossover (101).

Management depends on severity. Mild spasm often responds to additional sedation, analgesia, or intra-arterial vasodilators, while minimizing catheter manipulation (34). Moderate to severe spasm may require repeated vasodilator dosing, systemic nitroglycerin, forearm warming, lubricating agents such as Rotaglide or ViperSlide, or flow-mediated dilation using transient blood pressure cuff inflation. In refractory cases, ultrasound-guided radial nerve block or even general anesthesia may be necessary (34).

### 10.4 Radial artery occlusion

RAO is one of the most common postprocedural complication of TRA. A meta-analysis of 66 studies involving more than 31,000 patients reported incidence rates ranging from <1% to as high as 33%, depending on study methodology and timing of assessment (103). RAO occurs when endothelial injury from sheath insertion and catheter manipulation, combined with postprocedural compression and local stasis, promotes thrombus formation and subsequent arterial occlusion. Although RAO is usually clinically silent and rarely results in critical hand ischemia, preservation of radial artery patency is important for future vascular access and for use as a conduit in coronary bypass grafting (34).

Risk factors include female sex, advanced age, diabetes, and low body mass index, as well as procedural factors such as large sheath-to-artery ratios, inadequate anticoagulation, prolonged or occlusive hemostasis, multiple puncture attempts, and RAS (32, 95, 97, 104). Larger sheaths or guide catheters are associated with greater vascular injury and flow reduction, whereas smaller-caliber systems lower risk (32). Prolonged radial compression exceeding four hours strongly predicts RAO, highlighting the importance of patent hemostasis and achieving hemostasis with the shortest feasible duration (105). Randomized data show that hydrophilic coating or sheath length does not significantly influence RAO incidence (32). Operator experience and access-site proficiency also influence outcomes, as real-world data have demonstrated higher rates of access-site complications when operators perform less frequently utilized access techniques (106).

RAO is frequently asymptomatic and may go undetected without dedicated assessment (34). A palpable radial pulse does not reliably exclude RAO, as collateral flow may mask occlusion. Radial artery patency should therefore be routinely assessed before discharge and at follow-up, with duplex ultrasonography serving as the diagnostic standard (34).

Most cases require no intervention due to absence of symptoms, and spontaneous recanalization is common. A meta-analysis of 66 studies reported RAO in 7.7% of patients within 24 h of TRA, decreasing to 5.5% at one month (103). The randomized PROPHET-II trial similarly showed RAO rates of 13.9% immediately after hemostatic band removal, decreasing to 4.3% at 24 h and 3.0% at 30 days with patent hemostasis protocols (93). It should be emphasized that RAO demonstrates a well-documented tendency toward spontaneous recanalization over time, particularly when detected early and managed conservatively. Accordingly, treatment is generally reserved for symptomatic patients or in cases where preservation of the radial artery is clinically relevant (e.g., future conduit use or repeat access).

Treatment may include systemic anticoagulation, ipsilateral ulnar artery compression to augment radial flow, or, in select cases, percutaneous revascularization (34). A randomized study demonstrated that ipsilateral ulnar artery compression combined with 5,000 units of unfractionated heparin significantly reduced RAO rates (from 4.1% to 0.8%). In this protocol, a TR Band was inflated with 18 mL of air and positioned over the ipsilateral ulnar artery for one hour, followed by reassessment of radial patency. This strategy enhances radial flow through collateral recruitment and appears particularly effective when implemented early after occlusion detection (92).

Preventive strategies remain the cornerstone of RAO management and are mechanistically directed at preserving radial artery flow and minimizing endothelial injury. Strategies include minimizing sheath size relative to artery diameter to reduce luminal compromise and vascular trauma, ensuring adequate anticoagulation to prevent thrombus formation, shortening compression duration to limit flow stagnation, and applying patent hemostasis techniques to maintain antegrade flow during hemostasis. Use of sheathless guide catheters may offer further protection by reducing sheath-to-artery mismatch and vessel injury, with randomized comparisons showing a trend toward lower RAO rates compared with slender sheath systems, although data remain limited (34, 107).

Intraprocedural anticoagulation is also essential to prevent RAO, which is associated with inadequate anticoagulation. Lower ACT levels have been independently associated with higher RAO risk (108). UFH effectively reduces RAO in a dose-dependent manner, with intraarterial and intravenous routes proving comparably effective (3). A randomized study comparing high-dose UFH (100 IU/kg) with low-dose UFH (50 IU/kg) demonstrated a significantly lower rate of RAO with high-dose UFH (95). Alternatives such as enoxaparin and bivalirudin have demonstrated similar efficacy to UFH (109). In patients on therapeutic oral anticoagulation, adjunctive heparin appears safe and may further reduce RAO risk, although evidence remains mixed (110, 111).

### 10.5 Arterial pseudoaneurysm

Although rare (0.02%–0.09%), radial pseudoaneurysm warrants prompt recognition due to the potential for progressive enlargement or infection (14, 34, 112). It occurs when penetrating arterial injury, often from catheter or sheath insertion, results in a pulsatile extraluminal hematoma that communicates with the arterial lumen and is contained by surrounding tissue (34). The incidence is substantially lower than that observed with femoral access, further supporting the safety profile of TRA (113). Pseudoaneurysm should be suspected when a pulsatile swelling develops at the access site following TRA. Most cases are painless, though some patients may report local discomfort (34).

Risk factors include multiple puncture attempts, aggressive anticoagulation, large sheath size, inadequate compression after sheath removal, infection, and advanced age (97, 114). Delayed bleeding due to insufficient hemostasis may also contribute to pseudoaneurysm formation. Duplex ultrasonography remains the diagnostic modality of choice, demonstrating the characteristic “to-and-fro” flow pattern (34).

Management is typically conservative, with manual compression using a radial band or ultrasound-guided compression often sufficient (3, 34). For persistent pseudoaneurysms, ultrasound-guided thrombin injection may be performed, while surgical repair is rarely required and is reserved for large, symptomatic, or infected lesions (34). Prognosis is excellent when pseudoaneurysms are promptly recognized and appropriately managed.

### 10.6 Arteriovenous Fistula

AVF represents an abnormal communication between an artery and a vein, typically resulting from simultaneous penetration of both vessel walls during vascular access (3, 34). Radial AVF is exceedingly uncommon, with reported incidence up to 0.1% (3, 14, 34). In the RIVAL trial, which included 3,507 TRA patients, no cases were identified (14). A large retrospective study of 10,676 TRA patients reported an incidence of 0.08% (112).

The primary risk factor is multiple puncture attempts. The superficial venous anatomy surrounding the radial artery predisposes to inadvertent venous puncture during multiple access attempts (32, 97). Clinical presentation usually includes localized pain, swelling, or persistent access-site discomfort. A palpable thrill or audible bruit on examination should raise suspicion, and the diagnosis can be confirmed with duplex ultrasonography (3, 34).

Most radial AVFs are small and self-limiting. Conservative management with observation or prolonged compressive bandaging is appropriate in the majority of cases (3, 34). Intervention is reserved for patients who develop symptomatic arterial steal syndrome, venous congestion of the extremity, or high-output cardiac physiology. Treatment options include endovascular embolization, covered stent placement, or surgical repair with ligation, excision, or vessel reconstruction (3, 34, 115). Prognosis is generally favorable, as most lesions resolve without long-term sequelae when promptly recognized and appropriately managed (3, 34).

### 10.7 Compartment syndrome

Compartment syndrome is a rare but potentially devastating complication of TRA, with a reported incidence of 0.004% to 0.05% (14, 116). It occurs when a large forearm hematoma increases intracompartmental pressure, compromising both radial and ulnar arterial flow, leading to ischemia. If untreated, this can result in severe neurological injury and permanent functional impairment (34).

Risk factors include radial artery perforation, failed hemostasis at the puncture site, improper position of compression band, radial artery dissection, use of GPI, failure of early recognition, and low body mass index (97, 117). Diagnosis requires a high index of suspicion and experienced staff, particularly in patients with an enlarging forearm hematoma accompanied by disproportionate pain or evolving neurologic symptoms. Classic clinical signs include pain out of proportion, pulselessness, pallor, paresthesia, and paralysis (34).

Management requires urgent recognition and intervention. Early control of hematoma formation through appropriate compression and reversal of anticoagulation, when feasible, can prevent progression. Once compartment syndrome is developed, prompt fasciotomy is essential to preserve limb function and prevent irreversible ischemic and neurologic damage (34). With timely recognition and prompt fasciotomy, most patients recover full or near-normal limb function (118). Delayed diagnosis remains the principal determinant of adverse outcome. Delayed diagnosis, however, markedly increases the risk of irreversible ischemic and neurologic injury, including persistent deficits or contracture, and in severe cases may lead to systemic complications or death (119, 120).

### 10.8 Hand ischemia

Hand ischemia is a rare but serious complication of TRA, with a reported incidence of <0.2% (121). It is characterized by loss of Doppler signals in the ipsilateral radial artery and palmar arch, accompanied by impaired collateral flow through the ulnar artery on ultrasound. Pathophysiology includes embolization of thrombus from the radial artery and thrombosis formation of collateral vessels, often precipitated by vasospasm (97). This distinction has therapeutic implications, as embolic occlusion of distal vessels may not be corrected by proximal revascularization, whereas thrombotic or vasospastic mechanisms may respond to medical therapy with vasodilators and anticoagulation (121).

Patient- and procedure-related risk factors include smoking, peripheral artery disease, hypercoagulable states, Raynaud's disease, shock requiring vasopressor support, and RAS (97). Importantly, abnormal preprocedural Allen's test or other noninvasive assessments of collateral circulation do not reliably predict ischemic events, as compensatory recruitment of the ulnar circulation may occur acutely during catheterization (97). Consequently, routine Allen's testing is no longer recommended as a screening tool to exclude patients from TRA.

Clinical presentation ranges from mild ischemic changes to severe, limb-threatening ischemia. In mild cases, medical management with intra-arterial vasodilators such as verapamil, combined with systemic therapy using intravenous diltiazem and low-dose UFH, has been shown to restore palpable radial and ulnar pulses (97, 121). Severe cases, particularly those involving global ischemia of the hand, often require urgent surgical intervention (97, 121). Despite intervention, advanced ischemia carries a poor prognosis and is associated with high risk of tissue loss or amputation (121).

### 10.9 Persistent postprocedural pain

Acute postprocedural arm pain is not uncommon after TRA, occurring in approximately 5% of patients following PCI (105). In a retrospective study of 1,706 TRA patients, factors independently associated with postprocedural arm pain at discharge included hemostatic compression time greater than four hours, RAO, radial artery diameter <2.8 mm, and multiple puncture attempts (105).

While most postprocedural pain is transient, a subset of patients may develop chronic pain that persists beyond the immediate recovery period. A prospective study of 161 patients undergoing elective PCI reported persistent access-site pain in 3.7% of patients at three months of follow-up (122). Predictive factors for chronic pain included diabetes mellitus, pre-existing hand neuropathy, local hematoma formation, higher pain intensity within the first 48 h, and persistence of pain at 12, 24, and 48 h after PCI (122). As RAO may contribute to postprocedural pain, assessment for RAO should be incorporated into the diagnostic work-up (34). Given the association between RAO and postprocedural pain, routine postprocedural Doppler assessment may be justified in symptomatic patients.

Although the long-term prevalence of persistent pain is relatively low, these findings emphasize the importance of careful technique, optimal hemostasis, and early recognition of risk factors to minimize both acute and chronic pain after transradial procedures.

### 10.10 Neurological deficit

Neurological injury following TRA is exceedingly rare, as the radial artery at the typical puncture site is anatomically separated from the major nerves innervating the hand. Nonetheless, localized sensory disturbances such as tingling, numbness, or transient paresthesia lasting several days are not uncommon (32). A systematic review reported nerve damage in three studies with a combined incidence of 0.16%, while five studies reported sensory loss, tingling, or numbness with a pooled incidence of 1.52% (123).

Pathophysiology is most often related to extrinsic compression from a local hematoma or direct mechanical irritation during access attempts. Ultrasound can assist in identifying compressive hematoma, while nerve conduction studies may help confirm slowed conduction velocities in affected nerves (32).

Most neurological symptoms are transient and resolve spontaneously within days to weeks. For persistent or functionally limiting symptoms, treatment options include nonsteroidal anti-inflammatory drugs, temporary immobilization, local corticosteroid injections, physical therapy, or pharmacologic agents such as selective serotonin reuptake inhibitors (32).

### 10.11 Infection

Infection at the radial access site is rare, and data on its incidence are limited, as large randomized trials of TRA for CAG and PCI have not specifically reported infection-related outcomes. In a retrospective series of more than 1,500 neurointerventional TRA procedures, the access-site infection rate was 0.1% (124). Infection is recognized as a potential risk factor for radial artery pseudoaneurysm (34).

Most available evidence on access-site infection derives from settings in which catheters or sheaths remain in place for prolonged periods, such as arterial lines for hemodynamic monitoring, rather than from short-duration coronary interventions (125). Short procedural duration during coronary interventions likely explains the extremely low infection rates compared with prolonged arterial catheterization settings. Rare case reports of mycotic aneurysm highlight that, despite its rarity, infection can have serious consequences (126).

## 11. Current challenges

Although TRA has become the default vascular access strategy, several important challenges persist. In stable CAD, high-quality evidence is limited, as most randomized data are derived from ACS populations, restricting the ability to determine whether the benefits observed in higher-risk cohorts extend to lower-risk elective PCI. The optimal anticoagulation strategy during TRA is also not well examined. While TRA lowers access-site bleeding, the appropriate ACT range is not well established, and the bleeding advantage of alternative agents over UFH may be diminished in this setting, where baseline bleeding risk is relatively low.

Emerging access alternatives such as dTRA and transulnar access offer potential benefits, including lower rates of RAO and preservation of the proximal radial artery for future use as a coronary artery bypass graft conduit. However, these approaches are associated with higher crossover rates, longer cannulation times, and limited operator experience, requiring a learning curve similar to the initial adoption of TRA. A prior study suggests that approximately 200 cases are required to achieve stable success with distal TRA (127). Despite growing adoption, distal radial access remains an evolving technique that has not yet been fully standardized across operators and institutions.

Procedural complications of TRA, while generally infrequent, remain clinically relevant. Ultrasound-guided vascular access is increasingly recognized as an evolving standard for radial access, particularly proximal TRA, with the potential to improve first-pass success and reduce access-site complications. Although risk factors have been identified, their translation into individualized prevention strategies is still limited.

Moreover, procedural success continues to depend on operator expertise and institutional experience. Real-world registry data suggest that operators with the highest proficiency in radial access may have worse outcomes when performing femoral access procedures, including higher rates of access-site complications (106). Timely TRA access is essential in ACS cases, highlighting the importance of operator experience. TRA can be technically challenging in patients with small or tortuous vessels, and complex interventions requiring large-bore access may still necessitate femoral access, underscoring the ongoing need for tailored access-site selection.

## 12. Future directions

Future research should prioritize high-quality, dedicated studies in patients with stable CAD undergoing TRA. Large, multicenter randomized trials are needed to determine whether the benefits observed in ACS population translate to lower-risk elective PCI. Further investigations into anticoagulation during TRA, including ACT-guided dosing protocols and direct comparisons of UFH with alternative agents, are also warranted to refine practice in the transradial era, where baseline bleeding risk is inherently reduced.

Emerging alternatives such as dTRA and transulnar access require further evaluation in experienced centers, once the procedural learning curve has been overcome, to establish their role relative to pTRA. These studies may help clarify whether the potential benefits of these approaches, such as preservation of the proximal radial artery and reduced RAO, can be realized in routine practice without compromising procedural efficiency.

Personalization of vascular access represents another important future direction. Incorporating patient-level predictors of complications into procedural planning may enable more tailored prevention of RAO, RAS, and other access-site events. Advances in imaging modalities, risk stratification tools, and post-procedural monitoring are likely to play a key role in supporting this individualized strategy.

## 13. Conclusion

The TRA has transformed CAG and PCI, offering significant reductions in bleeding and vascular complications while improving patient comfort, earlier discharge and outcomes, particularly in ACS. Despite its widespread adoption, important challenges remain, including limited evidence in stable CAD, uncertainties surrounding optimal anticoagulation strategies, and technical considerations with emerging alternative access sites. As practice continues to evolve, future research should focus on generating robust evidence in lower-risk populations, refining anticoagulation protocols specific to TRA, standardizing definitions and assessment of RAO, and exploring the role of dTRA and ulnar approaches once procedural expertise matures. The incorporation of routine ultrasound-based radial artery follow-up in clinical trials may improve detection of subclinical RAO and better inform preventive strategies. In addition, emerging technologies such as ultrasound-guided access and AI-assisted procedural planning hold promise for improving access success and reducing complications. Advances in personalized access-site selection and personalization of anticoagulation strategy informed by patient-level risk stratification and supported by contemporary imaging and monitoring, are expected to further enhance safety, efficacy, and long-term vascular preservation in the transradial era.
